# Risk of Cardiovascular Disease Related to Metabolic Syndrome in College Students: A Cross-Sectional Secondary Data Analysis

**DOI:** 10.3390/ijerph16193708

**Published:** 2019-10-01

**Authors:** Insil Jang, Ji-Su Kim

**Affiliations:** 1 Department of Nursing, University of Ulsan, Ulsan 44610, Korea; 2 Department of Nursing, Chung-Ang University, Seoul 06974, Korea

**Keywords:** metabolic syndrome, risk factors, students, young adults, body mass index

## Abstract

Early detection of metabolic syndrome (MS) in young adults can lead to decreased aggravation and help prevent diabetes and cardiovascular diseases. This cross-sectional study aimed to identify the prevalence of MS and its components in Korean college students and was based on the Korean National Health and Nutrition Examination Survey, which used a stratified multistage probability sampling design. In total, 6.5% male and 4.1% female students had MS; of these, 26.6% of male and 25.8% of female students presented with at least one MS component. Height, weight, body mass index (BMI), waist circumference (WC), systolic blood pressure (SBP), diastolic blood pressure (DBP), glucose, high-density lipoprotein cholesterol, and triglycerides were significantly higher in men than in women, and all of these variables showed significant differences according to BMI. As the BMI increased, the level of each anthropometric, biochemical, and clinical variable increased. Although only a few students in Korea had three or more risk factors, the proportion of college students with one risk factor for MS was relatively high. Therefore, educational and intervention programs should be conducted in college students with overweight or obesity so that they can change their lifestyle to reduce the risk of cardiovascular diseases and diabetes.

## 1. Introduction

Metabolic syndrome (MS) is defined as a clustering of interrelated metabolic abnormalities, including central obesity, insulin resistance, dyslipidemia, and hypertension [1]. This syndrome is a complex disorder that represents an important risk factor for cardiovascular disease and type 2 diabetes mellitus, which are known as lifestyle diseases. Cardiovascular disease is the leading cause of death, aside from cancer, in all adults globally. Screening to determine the prevalence of MS and its most common risk factors in young adults is critical since initial studies have identified MS in young adults [2].

MS is characterized by race, region, sex, age, and family history; the results of previous studies are varied [3,4]. Furthermore, the prevalence of MS has been continuously increasing in the global population in recent decades, especially in the USA and Asia, due to the rapid Westernization of lifestyle behaviors, such as eating habits [5,6].

MS prevalence is estimated to increase with age; the prevalence is 20–25% in adults and 0.6–13% in college students in the USA [7,8,9]. Findings from the Young Adult Health Risk Screening Initiative study, conducted on 2722 college students aged 18–24 years, showed that 3% and 10% of female and male participants, respectively, had MS, and 54% and 77% of female and male participants, respectively, had at least one risk factor for MS [7]. Huang et al. [10] reported that 26–40% of students at the University of Kansas had at least one metabolic risk factor for MS, and no sex differences in the risk factors were noted, with the exception of glucose metabolism. A total of 1.7% of college students had MS, and most students with MS risk factors were men (58.3%) or had obesity (25%); additionally, 30.4% of all participants presented with at least one metabolic risk factor [11]. Fernandes and Lofgren [2] noted that 3.7% of the total students had MS, with more women (4.7%) than men (1.6%) and more students with obesity (38.5%) than with under/normal weight (0%) and overweight (5.7%).

Based on the 2015 statistics of the National Health Insurance Corporation (NHIC) in Korea, the rate of MS among adults over 30 years was 24% (27.3% men and 20.2% women). The morbidity of people with MS between the ages of 20 and 24 years was 3.9% (6% men and 2.3% women) and was 7.1% (10.8% men and 2.5% women) among people aged 25–29 years [12]. However, 25–26.3% of young adult participants had at least one risk factor, indicating that management is necessary for future health status [12].

Recently, one study was conducted to assess different population groups [11]. During college years, young people develop autonomy in their decision-making and choose their lifestyle habits which can strongly affect their future health. This is the perfect time to start choosing and adapting healthy lifestyles. In Korea, college students are increasingly stressed as they have just completed the academic burden of adolescence, with additional new burdens associated with their jobs and career paths [13]. If poor lifestyle habits are developed during this time, individuals will most likely continue to practice these habits until adulthood that can negatively impact an individual’s health [2]. Therefore, there is a need to investigate MS incidence in young adults. The earlier MS and its risk factors are identified, more is time available to encourage healthy lifestyle habits and provide health education [11,14]. Furthermore, efforts for early detection of MS in young adults can lead to targeted interventions and reduce the risk of future development of MS, which can eventually lead to diabetes and cardiovascular disease later in life.

This study aimed to identify the prevalence of MS and its components in Korean college students. Additionally, we aimed to determine the association between these components and sex and body mass index (BMI).

## 2. Methods

### 2.1. Study Design and Population

This study was based on the Korean National Health and Nutrition Examination Survey (KNHANES), a cross-sectional and nationally representative survey conducted by the Korea Center for Control and Prevention (KCDC) from 2010 to 2014. The survey used a stratified multistage probability sampling design by assigning weights to each respondent to obtain an equal probability, enabling the results to represent the entire Korean population. KNHANES comprises a health interview, a health behavior survey, a nutritional survey, and a health examination study. In this study, we examined the data of 1310 college students who were currently in or temporarily absent from college or university from 2010 to 2014.

### 2.2. Research Variables

#### 2.2.1. Socio-Demographic Variables

The following socio-demographic characteristics were recorded: age, sex, economic status, work situation, marital status, smoking status, monthly alcohol consumption, and regular exercise. The economic level of the household was classified based on the equivalent income (average monthly household income/$\sqrt{\mathrm{number}\mathrm{of}\mathrm{family}\mathrm{members}}$); the values making up the lowest 25% of the data were designated as part of the 1st quartile, and those in the subsequent three levels (25% each) were designated as the 2nd, 3rd, and 4th quartiles. Employment and marital statuses were categorized as “Yes” and “No” for those who were currently working versus not working and for those who were married versus were single or were separated from their spouses due to death or divorce. Smoking status was categorized based on whether the participants had never smoked, had smoked in the past, or were currently smoking. Alcohol intake was based on a person’s drinking patterns during the past month. Regular exercise was defined as a strenuous or moderate physical activity performed for at least 20 min in one session, three times a week.

#### 2.2.2. Anthropometric Variables

Anthropometric measurements were similarly conducted by well-trained examiners during the study periods. The height, waist circumference, and weight were measured to the nearest 0.1 cm and 0.1 kg using a portable stadiometer (Seca 225, Seca, Hamburg, Germany); a ruler (Seca 200, Seca, Hamburg, Germany), which was used at the end of expiration, from the narrowest point between the lower borders of the rib cage and the iliac crest; and a calibrated balance-beam scale (GL-6000-20; G-tech, Seoul, Korea), respectively.

#### 2.2.3. Biochemical Variables

After a 12-hour overnight fast, venous blood samples were collected and immediately sent to a central, certified laboratory, and the plasma was separated immediately through centrifugation. The fasting plasma concentrations of glucose and lipids were measured using an enzymatic and hexokinase UV assay in a central laboratory using a chemistry analyzer (Hitachi 7600, Tokyo, Japan).

#### 2.2.4. Clinical Variables

Subjects were asked to refrain from smoking or consuming caffeine before the measurements. Systolic and diastolic blood pressure was measured twice, using a mercury sphygmomanometer (Baumanometer; Baum, Copiague, NY, USA), in a sitting position and after resting for at least 10 min.

#### 2.2.5. The MS Criteria

Based on the National Cholesterol Evaluation Program for Adult Treatment Panel III [15], an individual may be diagnosed with MS if they have three or more of the following criteria: (1) waist circumference >90 cm in men and >80 cm in women using the International Obesity Task Force criteria for the Asia-Pacific population [16]; (2) triglycerides ≥150 mg/dL or medication use; (3) high-density lipoprotein (HDL) cholesterol <40 mg/dL in men and <50 mg/dL in women or medication use; (4) blood pressure ≥130/85 mmHg or antihypertensive medication use; and (5) fasting glucose ≥100 mg/dL or medication use (insulin or oral hypoglycemic agents).

### 2.3. Statistical Analysis

The SAS survey procedure (ver. 9.3; SAS Institute Inc., Cary, NC, USA) was used to run a complex sample design to analyze the survey data, ensuring appropriate sampling weights and nationally representative estimates. For the anthropometric, clinical, and biochemical variables, means and standard errors (SE), such as geometric means (95% confidence interval) or proportions, were calculated. If necessary, a logarithmic transformation was performed to achieve a normal distribution. For the statistical analyses, ANOVA, t-tests, and χ^2^-test were used to determine the association between the number of MS symptoms associated with BMI classification and sex. Inferential statistical analyses were considered significant if the *p*-value was <0.05.

### 2.4. Ethical Considerations

This study was a secondary analysis using the 2010–2014 KNHANES data and was reviewed and approved by the Institutional Review Board of the KCDC (Approval No. 2010-02CON-21-C, 2011-02CON-06-C, 2012-01EXP-01-2C, 2013-07CON-03-4C, and 2013-12EXP-03-5C). Informed consent was obtained from all of the participants when the 2010 to 2014 KNHANES were conducted by the KCDC.

## 3. Results

Of the 1310 college students enrolled, 50.3% were women, 69.6% were single, and 62.4% were aged between 20 and 24 years. Overall, age ranged between 18 and 66 years, with a mean of 23.4 years (SE: 0.2). Additionally, 58.5% exclusively studied, and the remaining 41.5% studied while working at the same time; 30% fell into the 4th quartile in terms of their economic status. Overall, 22.7% were currently smoking, 74.7% were drinking monthly, and 24.9% had a regular exercise (Table 1).

**Figure 1:**
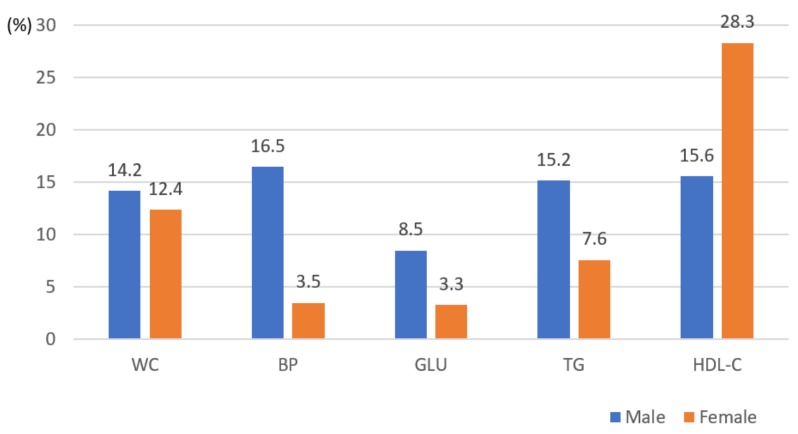
Association between individual metabolic syndrome components and sex (n = 1310). Abbreviations: WC, obesity reference with waist circumference (χ^2^ = 0.629, *p* = 0.427); BP, blood pressure (χ^2^ = 52.119, *p* < 0.0001); GLU, fasting glucose (χ^2^ = 13.063, *p* < 0.001); TG, triglycerides (χ^2^ = 13.267, *p* < 0.001); HDL-C, high-density lipoprotein cholesterol (χ^2^ = 24.028, *p* < 0.0001).

**Figure 2:**
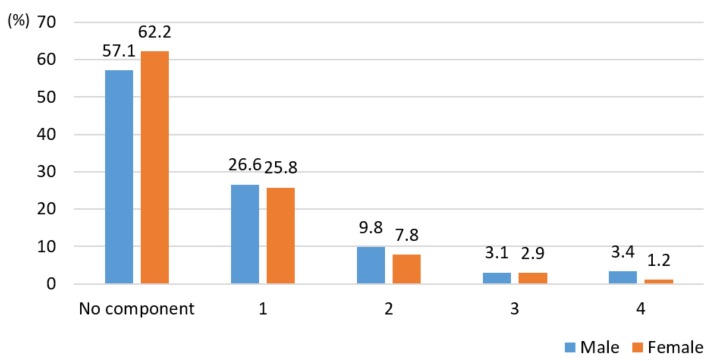
Association between the number of metabolic syndrome components and sex (n = 1310). Metabolic syndrome components: waist circumference, blood pressure, fasting glucose, triglycerides, and high-density lipoprotein cholesterol (χ^2^ = 7.502, *p* = 0.111).

Table 2 indicates sex differences in relation to anthropometric measures, biochemical, and clinical variables. The variables, such as height (t = 36.476, *p* < 0.0001), weight (t = 21.619, *p* < 0.0001), BMI (t = 8.439, *p* < 0.0001), waist circumferences (t = 15.936, *p* < 0.0001), systolic blood pressure (t = 14.866, *p* < 0.0001), diastolic blood pressure (t = 9.274, *p* < 0.0001), fasting blood sugar (t = 2.740, *p*=0.006), HDL cholesterol (t = 9.975, *p* < 0.0001), and triglycerides (t = 7.205, *p* < 0.0001), with the exception of low-density lipoprotein (LDL) cholesterol, were significantly higher in men than women (Table 2). Aside from waist circumferences indicating obesity, there were significant sex differences in hypertension (*p* < 0.0001), hyperglycemia (*p* < 0.0001), hypertriglyceridemia (*p* < 0.001), and HDL cholesterol (*p* < 0.0001) (Figure 1). The prevalence of MS in college students was 6.5% and 4.1% in men and women, respectively. Nevertheless, 26.6% and 25.8% of men and women presented with at least one MS symptom, while 16.3% and 12% of men and women presented with at least two MS risk factors, respectively (Figure 2).

**Figure 3:**
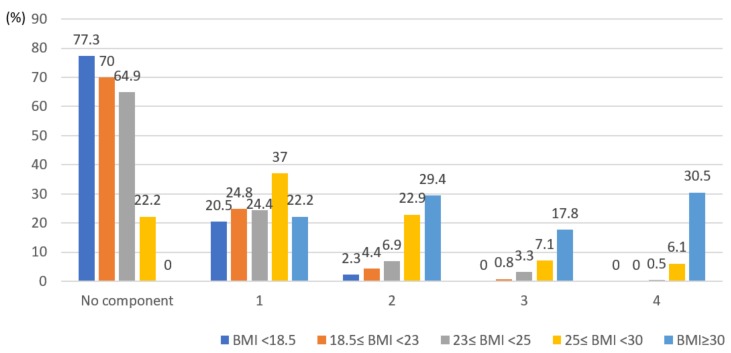
Association between the number of metabolic syndrome components and body mass index (n = 1310). Metabolic syndrome components: waist circumference, blood pressure, fasting glucose, triglycerides, and high-density lipoprotein cholesterol.

Table 3 indicates BMI classifications in relation to anthropometric measures, biochemical, and clinical variables. All variables, namely, height (*p* < 0.0001), weight (*p* < 0.0001), waist circumferences (*p* < 0.0001), systolic blood pressure (*p* < 0.0001), diastolic blood pressure (*p* < 0.0001), fasting blood sugar (*p* < 0.0001), HDL cholesterol (*p* < 0.0001), LDL cholesterol (*p* < 0.0001), and triglycerides (*p* < 0.0001), significantly differed based on the BMI classification. Specifically, as the BMI increased, the level of the anthropometric, biochemical, and clinical variables also increased (Table 3). Therefore, the number of individual MS risk factors also increased in this study (Figure 3).

## 4. Discussion

This study aimed to identify the prevalence of the MS and its risk factors in a Korean college student population. A total of 22.7% of the college students were currently smoking, 35.1% of whom were men; this was eight times more than that of women. Korea has the highest number of adult men who are smoking among the Organization for Economic Cooperation and Development countries with a reported rate of 39.3% despite efforts of the Ministry of Health and Welfare [17,18]. In contrast, the smoking rate for adult women was 5.5% [18]. Considering that the smoking rate does not significantly differ between college students and adult men, smoking habits are most likely continued from college years onward. Therefore, the difference in the smoking rate with regard to sex could impact MS morbidity. In 2015, the drinking rate among college students surveyed was high at 74.7% (82.2% men and 62.6% women), and that of Korean adults was 78.5% (86.6% men and 70.8% women); this rate increases every year [18]. Frequent drinking habits leads to overeating, which is related to obesity and also associated with an increased rate of MS. Furthermore, 75.1% of college students did not exercise regularly, and the rate of regular exercise was higher in men than women students. Therefore, college student lifestyles include frequent drinking and smoking and no regular exercise. College students choose these unhealthy lifestyles because they want to escape from the stress due to the control of their family, pressures in school, and influences of the surrounding environment, such as friends and seniors. These findings suggest that college students would benefit from various intervention programs to help prevent MS in Korea.

We found that anthropometric, biochemical, and clinical variables significantly differed by sex. BMI, waist circumferences, systolic blood pressure, diastolic blood pressure, fasting blood sugar, HDL cholesterol, and triglycerides, with the exception of LDL, were higher in men than in women. These results were similar to those of the previous studies in other countries [2,7,10,11,19,20].

The prevalence of MS in college students was 5.3% (6.5% men and 4.1% women). The prevalence rate of students with MS in this study was higher than that mentioned in some studies [2,7,10,11] and lower than that mentioned in others [19,20,21]. These data show that the proportion of college students with MS in Korea is higher than that in Western countries. Generally, researchers ignore young adults with chronic disease development in their risk analyses, even when assessing MS prevalence. Diagnostic assessment of these individuals is needed for the early identification of MS and its components [2,22]. In many studies, MS morbidity varies based on race, region, sex, age, and participant lifestyle choices; thus, the rate and risk factors of MS should be compared with the various characteristics of the participants. Additionally, further research based on the KNHANES data is needed to identify the trend in changes associated with MS and its risk factors in Korea. Nevertheless, 26.6% and 25.8% of men and women college students, respectively, presented with at least one component for MS. These results were similar to those of the 2015 NHIC statistics in Korea and lower than the range of 27–37% reported in other studies [2,7,10,11,12]. Although many students in Korea have only one component of MS, the proportion of studies with three or more components was relatively high. Therefore, the prevalence of MS among Korean college students should be highlighted so that educational and intervention programs to create awareness around lifestyle changes are implemented to reduce the prevalence of MS and its components.

Particularly, the association between the MS components and sex, hypertension, hyperglycemia, hypertriglyceridemia, and low HDL cholesterol, with the exception of a waist circumference indicative of obesity, was higher in men than in women. These results were similar to those of the previous studies conducted in other countries [7,10,11,19,20]. Specifically, men had a 61% increase in the prevalence of MS, meaning the prevalence of MS was 1.56-times as that observed in women. Similarly, some experts identified higher MS prevalence rates in men, but some studies showed only slight sex differences [7,10,11,12]. Furthermore, some studies identified a distinct relationship between MS and sex, with higher proportions among women [2,21,22,23,24]. This diverse outcome variability is due to the fact that the syndrome is highly affected by the ethnic background and lifestyle of students. Several studies have supported the fact that Caucasians and African-Americans have a lower rate of MS prevalence than other ethnic groups, including Mexican-American, Hispanic, and Asian [2,10,24,25]. Thus, the long-term trends of MS based on sex, age, and ethnic background should be confirmed. Furthermore, guidelines for each culture using country-specific data to manage MS should be developed.

Finally, all data on risk factors of MS, such as waist circumferences, systolic blood pressure, diastolic blood pressure, fasting blood sugar, HDL cholesterol, LDL cholesterol, and triglycerides, significantly differed based on the BMI classification in college students. As the BMI increased, the levels of anthropometric, biochemical, and clinical variables and the number of individual components of MS increased. Among the students without any MS components, 77.3% were underweight, and 70% had a normal weight. However, 77.8% of students with obesity (25≤BMI<30 kg/m^2^), based on the Asian BMI classification, had one or more components, and 13.2% had MS. All students who belonged to the severely obese group (BMI≥30 kg/m^2^) had one or more risk factors for MS, 48.3% of whom had the syndrome. These findings indicate that BMI, including waist circumference, is a crucial determinant of MS. Based on the previous studies, the rate of obesity was higher among students with some MS components [11,21,24,26]. The causes of MS are not well known, but insulin resistance is generally a fundamental problem contributing to the syndrome. Insulin resistance is associated with central obesity and is directly linked to obesity. Based on the 2015 KNHANES data, 36.8% of students had obesity, with a BMI of over 25 kg/m^2^, demonstrating that the degree of obesity in young adults in Korea was similar to that in other countries [2,7,11,19,23,24] and that the rate of obesity among twenty-year-old individuals was high [18]. Unhealthy lifestyles and lack of exercise are the main causes of obesity in college students [27]. Specifically, the number of students with overweight or obesity should not be overlooked, and lifestyle modifications should be implemented to reduce the risk of cardiovascular disease and diabetes. Thus, weight management is crucial for college students with obesity with MS and its components, and diet and exercise programs should be implemented in Korea. It is also interesting to note that approximately 20% of college students who had underweight and normal weight had at least one MS component. Therefore, there is a need to extend health-related education focused only on the obese group rather than on the underweight or normal weight group for preventing cardiovascular disease due to developing MS.

This study had some limitations. First, secondary data were analyzed; therefore, there were restrictions based on data inclusion regarding diverse characteristics affecting the prevalence of MS and its components. In addition, college students were defined as those who were currently in or temporarily absent from college or university in our study. We could not use diverse characteristics of college students, such as their grades and major, eating habits, and sedentary lifestyle habits associated with computer and social media use. Therefore, further studies will be needed to assess factors affecting MS and various characteristics of college students in more detail.

## 5. Conclusions

Only a few students in Korea had three or more risk factors, while the proportion of college students with one risk factor for MS was found to be relatively high. In addition, overweight and obesity were associated with a high frequency of individual components of MS.

This study supports the need to continue screening for all MS risk factors (clinical and demographic) in the college population to prevent the prevalence of chronic diseases in Korea. Furthermore, different intervention programs that allow for lifestyle modifications, such as diet, exercise, smoking cessation, and alcohol consumption, should be recommended by the government to avoid obesity, MS, and its associated risk factors.

## Figures and Tables

**Table 1:** Demographic characteristics (n = 1310).

Variables		Total Mean ± SE, n (%)	Male Mean ± SE, n (%)	Female Mean ± SE, n (%)
Age		23.4 ± 0.2	23.5 ± 0.2	23.3 ± 0.3
	18–19	239 (16.8)	107 (12.4)	132 (23.9)
	20–24	756 (62.4)	369 (64.0)	387 (59.8)
	25–66	315 (20.8)	175 (23.5)	140 (16.3)
Economic status	1	146 (13.2)	67 (11.7)	79 (15.7)
	2	344 (28.0)	180 (28.3)	164 (27.5)
	3	385 (28.8)	203 (30.7)	182 (25.7)
	4	420 (30.0)	196 (29.4)	224 (31.0)
Work situation	Only studies	771 (58.5)	382 (58.7)	389 (58.2)
	Studies/workers	539 (41.5)	269 (41.3)	270 (41.8)
Marital status	Single	231 (69.9)	109 (74.6)	122 (63.8)
	Married	152 (30.1)	53 (25.4)	99 (36.2)
Smoking status	Nonsmoker	980 (74.6)	367 (61.5)	613 (94.4)
	Ex-smoker	32 (2.6)	23 (3.5)	9 (1.3)
	Current smoker	233 (22.7)	207 (35.1)	26 (4.3)
Monthly drinking	No	380 (25.3)	116 (17.8)	264 (37.4)
	Yes	925 (74.7)	534 (82.2)	391 (62.6)
Regular exercise	No	819 (75.1)	380 (70.1)	439 (83.3)
	Yes	240 (24.9)	151 (29.9)	89 (16.7)

Abbreviations: SE, standard errors.

**Table 2:** Distribution of means for anthropometric and biomarker (n = 1310).

Variables	Total	Sex		t	*p*
		Male (n = 651)	Female (n = 659)		
	Mean ± SE	Mean ± SE	Mean ± SE		
Height	169.5 ± 0.3	174.5 ± 0.3	161.5 ± 0.3	36.476	<0.0001
Weight	65.3 ± 0.5	71.3 ± 0.6	55.8 ± 0.4	21.619	<0.0001
BMI	22.6 ± 0.1	23.4 ± 0.2	21.4 ± 0.2	8.439	<0.0001
WC	76.6 ± 0.3	80.2 ± 0.4	70.8 ± 0.4	15.936	<0.0001
SBP	110.4 ± 0.4	114.2 ± 0.5	104.3 ± 0.5	14.866	<0.0001
DBP	71.9 ± 0.3	73.9 ± 0.4	68.6 ± 0.4	9.274	<0.0001
Glucose	88 ± 0.3	88.5 ± 0.4	87.0 ± 0.4	2.740	0.0063
Total Cholesterol	170.4 ± 1	169.9 ± 1.4	171.2 ± 1.3	0.678	0.4967
HDL-C	52.7 ± 0.4	50.2 ± 0.5	56.8 ± 0.5	9.975	<0.0001
LDL-C	98.8 ± 0.9	99.0 ± 1.3	98.5 ± 1.2	0.332	0.7369
Triglycerides	82 (79.3–84.7)*	89.6 (85.5–93.9)*	71.1 (68.3–74.1)*	7.205	<0.0001

* Geometric mean (95% CI). Abbreviations: BMI, body mass index; WC, waist circumference; SBP, systolic blood pressure; DBP, diastolic blood pressure; HDL-C, high-density lipoprotein cholesterol; LDL-C, low-density lipoprotein cholesterol.

**Table 3:** Association between metabolic syndrome-related components and BMI classifications (n = 1310).

Variables	BMI					F	*p*
	<18.5 (n = 155)	18.5≤ <23 (n = 673)	23≤ <25 (n = 209)	25≤ <30 (n = 220)	30≥ (n = 53)		
	Mean ± SE	Mean ± SE	Mean ± SE	Mean ± SE	Mean ± SE		
Height	166.4 ± 0.6	168.8 ± 0.4	171.0 ± 0.6	171.8 ± 0.6	169.7 ± 1.3	10.620	<0.0001
Weight	48.7 ± 0.4	59.5 ± 0.3	70.3 ± 0.5	79.6 ± 0.7	94.9 ± 1.9	501.290	<0.0001
WC	64.1 ± 0.4	72.4 ± 0.3	79.7 ± 0.4	87.3 ± 0.5	99.3 ± 1.4	528.330	<0.0001
SBP	105.0 ± 0.8	108.4 ± 0.5	111.5 ± 0.8	115.9 ± 0.9	119.9 ± 1.9	32.240	<0.0001
DBP	70.0 ± 0.7	70.3 ± 0.4	72.3 ± 0.7	75.1 ± 0.7	79.3 ± 1.7	14.640	<0.0001
Glucose	85.2 ± 0.6	86.7 ± 0.3	89.3 ± 0.8	89.3 ± 0.6	97.9 ± 1.9	17.510	<0.0001
Total Cholesterol	160.5 ± 2.3	165.1 ± 1.3	174.6 ± 2.2	181.1 ± 2.9	195.6 ± 5.6	16.580	<0.0001
HDL-C	57.4 ± 1.0	54.6 ± 0.5	52.0 ± 0.7	47.5 ± 0.7	43.8 ± 1.9	29.760	<0.0001
LDL-C	88.7 ± 1.9	93.8 ± 1.0	103.9 ± 2.1	109.3 ± 2.7	120.3 ± 5.0	19.680	<0.0001
Triglycerides	66.4 (61.5–71.6)*	74.3 (71.4–77.3)*	84.0 (78.1–90.2)*	104.7 (95.5–114.8)*	142.5 (120.4–168.6)*	27.660	<0.0001

* Geometric mean (95% CI). Abbreviations: BMI, body mass index; WC, waist circumference; SBP, systolic blood pressure; DBP, diastolic blood pressure; HDL-C, high-density lipoprotein cholesterol; LDL-C, low-density lipoprotein cholesterol.
